# Possible benefits for environmental sustainability of combined therapy with hemodialysis and peritoneal dialysis in Japan

**DOI:** 10.3389/fneph.2024.1394200

**Published:** 2024-11-14

**Authors:** Kei Nagai

**Affiliations:** ^1^ Department of Nephrology, Hitachi General Hospital, Hitachi, Ibaraki, Japan; ^2^ Department of Nephrology, Faculty of Medicine, University of Tsukuba, Tsukuba, Ibaraki, Japan

**Keywords:** hemodialysis, peritoneal dialysis, natural disaster, carbon footprint, capital investment

## 1 Introduction

Chronic kidney disease (CKD) progresses with aging and has a high prevalence in the elderly (1). As the proportion of elderly in the Japanese population continues to increase, the number of patients with end-stage kidney disease (ESKD) requiring maintenance dialysis is also growing (2). Dialysis care requires significant infrastructure and natural resources, and generates large amounts of waste (3–5). Sustainability of dialysis care is important because the equipment used for dialysis is not recyclable. Patients with ESKD are therefore vulnerable to resource shortages in the future, which is the reason why “Green Nephrology” has been established in numerous countries to develop environmentally friendly dialysis therapies and raise awareness of these environmental issues (6–12). Regarding dialysis modality, hemodialysis (HD) waste contains many infectious plastics such as dialysis membranes and dialysis circuits that must be incinerated and landfilled. Peritoneal dialysis (PD) waste at home, on the other hand, can be general waste disposal. Thus, it will be realized that there is a difference in the environmental impact of HD and PD in daily practice. In 2021, approximately 3.9 million people worldwide were treated with dialysis for ESKD. Currently, 89% of those undertaking dialysis receive HD and 11% receive PD (13). Even taking the issue of waste as one example, it is important to note the environmental aspect of whether HD or PD will be the treatment of choice around the world in the future.

Given the current state of dialysis care in Japan, which is highly dependent on in-center HD, it is difficult to imagine a dramatic increase in home-based PD in the absence of a proactive policy initiative. It is well known that aged Japanese people do not always want home treatment, preferring to be attended to by dialysis professionals at clinics and hospitals. In contrast, Japan has a history of combined PD and HD (PD+HD) as maintenance dialysis in practice (14) and PD+HD is also recognized as an insured procedure. The combined therapy is clinically relevant in Japan, as shown by the increase in patients receiving PD+HD therapy from 1683 patients (18.8% of PD patients) at the end of 2013 (15) to 1903 patients (19.2% of PD patients) at the end of 2019 (16). In principle, combined therapy had been performed only in facilities where both PD and HD can be treated. Following the reimbursement reform in 2020, each patient can receive PD and HD at separate medical facilities. That is, a patient can receive PD service at a clinic without HD beds, and weekly HD at a different HD facility that does not provide any PD services.

HD is conventionally used in combination with PD at the point when water and solute removal become insufficient by PD alone (16). It has been challenging to evaluate the scientific significance of the combined therapy because of the extreme difficulty of setting up a prospective randomized clinical trial (17). However, PD+HD therapy has benefits in terms of quality of life, for example, in enabling flexibility of lifestyle. Patients can enjoy leisure time by taking one or two days of PD rest a week while also interacting with healthcare providers by attending HD once per week. Low-frequency monthly visits for PD monotherapy are preferable for younger and working patients, whereas weekly HD visits may be more suitable for older patients who prefer constant monitoring of their health and PD treatment status. Although the evidence is presently inconclusive, we have experienced the advantages of the combined dialysis method in terms of sustainability of patient health through residual renal function, peritoneal function, and PD technique survival at a dialysis facility (18–20).

As yet, we have not been able to draw any conclusions about the environmental impact or sustainability of PD+HD combination therapy, or its superiority over HD, which is the mainstream treatment in Japan. In this opinion article, we will explore the theoretical advantages of combination therapy, which is widely used in Japan, in terms of sustainability.

## 2 Environmental sustainability in dialysis therapy

### 2.1 Carbon footprint in combination therapy of PD and HD

Greenhouse gases consist of several atmospheric gases responsible for rising temperatures, and include carbon dioxide and methane. The emissions of these gases can be calculated as the carbon footprint (CFP) for a particular industry, and should be reduced as much as possible. The healthcare sector generates substantial economic activity and is responsible for emission of greenhouse gases (21), and the large CFP of dialysis therapy has been previously noted (8). Although CFP studies are yet to be fully validated, the prevailing view is that HD has a large CFP, whereas PD and renal transplantation have small CFPs (22).

**Figure 1:**
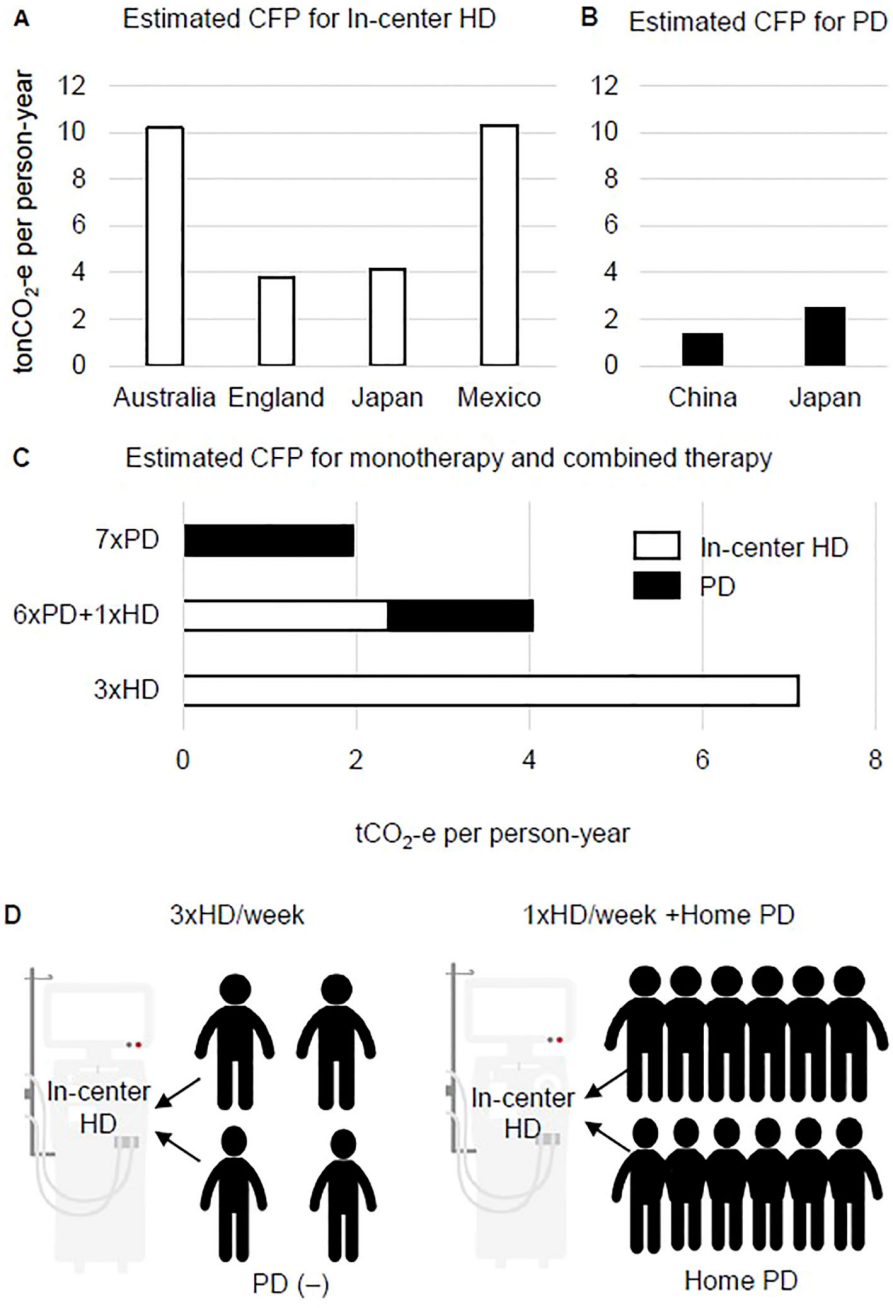
Putative benefit of combined therapy of hemodialysis and peritoneal dialysis on carbon footprint and dialysis console sharing. **(A)** Estimated carbon footprint (CFP) for in-center hemodialysis (HD) in four countries. **(B)** Estimated CFP for home-based peritoneal dialysis (PD) in two countries. **(C)** Estimated CFP for HD, PD, and the combination PD+HD therapy model based on the average of CFP values for HD and PD shown in **(A, B, D)**. In the weekly HD sessions of PD+HD combined therapy, a maximum of 12 patients can share the same console.

The CFP per person per year for in-center HD has been reported as 3.8 t in the UK (7), 10.2 t in Australia (8), 4.1 t in Japan (23), and 10.3 t in Mexico (24) (**Figure 1A**). For home PD, CFP has been reported as 1.4 t in China (9) and 2.5 t in Japan (25) (**Figure 1B**). Simple comparisons are difficult to make because these calculations include numerous methodological differences, for example, information regarding the original dialysis patients, the units of resource consumption used to calculate CFP, and the aspects of dialysis that are included (drugs, dialysis membranes, patient visits and staff transport) (26). Nevertheless, the average estimated CFP for HD in the four countries mentioned above was 7.9 t of carbon dioxide equivalent (tCO_2_-e), and the average for PD in the two countries mentioned above was 2.0 tCO_2_-e. Based on these estimations, the CFP would be 4.3 tCO_2_-e for combined treatment of HD once a week and PD six times a week (**Figure 1C**). The low CFP of PD monotherapy is clearly preferable, but environmental sustainability is better with PD+HD combination therapy than with in-center HD monotherapy (**Figure 1C**). However, it remains unclear whether combining the dialysis methods would enable the overall CFP to be reduced below that for HD monotherapy, as it is undeniable that patients must manage both blood access and peritoneal access in PD+HD combination therapy, and that access-related complications increase the environmental costs associated with hospitalization.

The total annual CFP of maintenance HD for one patient in Japan is estimated as 4.1 tCO_2_-e, which includes the production and maintenance of dialysis equipment (49.2 kgCO_2_-e), electricity (551.3 kgCO_2_-e), and drugs (2171.0 kgCO_2_-e); as well as transport by healthcare staff (142.3 kgCO_2_-e) and by patients for visits to the center (1109.7 kgCO_2_-e) (25). Among these items, the scope of this opinion article includes patient visits to hospital, which is the second largest contributor to the CFP of HD patients. If in-center HD is commonly performed, the relatively long hospital visits required for dialysis therapy add to the CFP and to environmental costs, particularly in medically underpopulated rural areas (27). As the distance between each patient’s place of residence and the hospital is fixed, reducing the number of hospital visits by utilizing PD+HD combination therapy should be prioritized in rural areas of Japan.

### 2.2 Concerns about excess carbon footprint for fixed capital investment in dialysis facilities

The CFP of healthcare shows a generally increasing trend. A breakdown of the contributing factors over time in Japan reveals the most notable change in fixed capital investment, which was estimated as 9.0 megatons CO_2_-e of the total 62.5 megatons CO_2_-e in 2015 (14.3%), but increased to 13.0 of the total 75.1 megatons CO_2_-e (17.3%) in 2020 (1, 28). No study has reported the precise CFP of HD console manufacture as a proportion of the total dialysis CFP. Over the past decades, HD has consistently accounted for 97% of maintenance dialysis in Japan. The number of dialysis consoles and the number of dialysis patients have increased from 606 consoles for 949 patients in 1970, 18,963 consoles for 36,397 patients in 1980, 40,723 consoles for 103,296 patients in 1990, 79,709 consoles for 206,134 patients in 2000, 118,622 consoles for 298,252 patients in 2010, and 147,358 consoles for 347,474 patients in 2022, and the number of patients sharing a console has remained steady, at 2.5 HD patients per console (29). Although the current number of HD consoles is the highest over time, it is inevitable that this number will decline at some point due to depopulation. In other words, increasing the production of HD consoles without future planning may result in a future surplus. As dialysis consoles typically last for 7–10 years, for maximum efficiency the number of consoles produced must take into account the anticipated number of HD patients in the future. The annual survey of dialysis patients conducted by the Japanese Society of Dialysis Therapy (JSDT) found that there were 349,700 chronic dialysis patients as of the end of 2021 and 347,474 as of the end of 2022 (29), which marks the first episode of a decreasing trend since the JSDT surveys began. This finding is consistent with the expected result of a decrease in new dialysis inductions and an increase in dialysis patient deaths due to an increase in the age of dialysis patients and a decrease in the general population (30). Unfortunately, the time to start reducing the number of dialysis consoles in Japan may already have passed.

Nevertheless, the situation of an insufficient number of HD consoles for the required HD treatments must be avoided. To ensure a steady supply of dialysis consoles without excesses or shortages, one solution in underpopulated areas is for more patients to share a console. Most in-center HD is designed as one dialysis bed and a console that is shared by four patients (four courses of dialysis: morning and afternoon starting on Monday, morning and afternoon starting on Tuesday) for three sessions per week. In the case of weekly HD, one bed can be maximally shared by up to 12 patients receiving PD+HD on Monday to Saturday mornings or afternoons (**Figure 1D**). In our experience, PD+HD combination therapy when PD efficiency and water removal are insufficient can be used for approximately 1.5 years before final conversion to HD monotherapy (31). Of note, PD+HD combination therapy has clinical significance as it enables peritoneal lavage to be performed approximately once a week by in-center support, and allows setup time for PD catheter removal during the waiting period and for teaching patients how to prepare for three sessions per week of HD. In addition, combination therapy appears to have economic benefits as it allows a single facility to treat more dialysis patients in the case of a limited number of dialysis consoles. In the event of a natural disaster that reduces the number of available HD beds, combination therapy might help facilities to cope by providing PD monotherapy for a several weeks in PD+HD patients. However, as the concept of console sharing is highly theoretical and beyond patients’ health problems and quality of life, there are substantial hurdles to be overcome before this model can be widely spread into practice.

## 3 Discussion

Treatment choices and prescriptions for dialysis are made with the expectation of clinical benefits such as improved life expectancy and quality of life. In this regard, the present opinion article does not consider the clinical significance of patient prognosis. At present, there is no clear evidence that PD+HD combination therapy improves life expectancy. In practice, however, there are certain benefits of home-based PD for continuing work and social activities. Combination PD+HD therapy offers “co-benefits” for both patients and the environment that are apparent as reductions in CFP and capital investment.

A disadvantage of PD+HD is the increased access (i.e., blood access and peritoneal access) to dialysis that the patient must manage. There is a risk of a higher rate of hospitalization events related to access problems for combined dialysis therapy that may in turn increase the CFP. Nevertheless, home-based PD and infrequent in-center HD is likely to be a better choice for the aging population because of the patient preference for medical assistance with moderately frequent (weekly) visits to hospital. Taken overall, the development of sustainable dialysis treatment in Japan, which has the most rapidly aging society in the world, might be supported by combining PD with HD once a week rather than shifting to conventional in-center HD three times per week.
